# A 20 m wide-field diffraction-limited telescope

**DOI:** 10.1098/rsta.2020.0141

**Published:** 2021-01-11

**Authors:** Ryker W. Eads, J. Roger P. Angel

**Affiliations:** ^1^ Department of Optical Sciences, University of Arizona, Tucson, AZ, USA; ^2^ Steward Observatory, University of Arizona, Tucson, AZ, USA; ^3^ Richard F. Caris Mirror Laboratory, University of Arizona, Tucson, AZ, USA

**Keywords:** moon-based, space telescope, diffraction-limited, four-mirror, wide field, UVOIR

## Abstract

A 20 m space telescope is described with an unvignetted 1° field of view—a hundred times larger in area than fields of existing space telescopes. Its diffraction-limited images are a hundred times sharper than from wide-field ground-based telescopes and extend over much if not all the field, 40 arcmin diameter at 500 nm wavelength, for example. The optical system yielding a 1°, 1.36 m diameter image at f/3.9 has relatively small central obscuration, 9% by area on axis, and is fully baffled. Several carousel-mounted instruments can each access directly the full image. The initial instrument complement includes a 400 gigapixel silicon imager with 2 µm pixels (0.005 arcsec), and a 60 gigapixel HgCdTe imager with 5 µm pixels (0.012 arcsec). A multi-object spectrograph with 10 000 fibres will allow spectroscopy with 0.02 arcsec resolution. Direct imaging and spectroscopy of exoplanets can take advantage of the un-aberrated, on-axis image (5 nm RMS wavefront error). While this telescope could be built for operation in free space, a site accessible to a human outpost at the Moon's south pole would be advantageous, for assembly and repairs. The lunar site would allow also for the installation of new instruments to keep up with evolving scientific priorities and advancing technology. Cooling to less than 100E K would be achieved with a surrounding cylindrical thermal shield.

This article is part of a discussion meeting issue ‘Astronomy from the Moon: the next decades (part 1)’.

## 1. Background

Existing and currently planned large space telescopes have been designed to exploit the unique advantages of space—freedom from atmospheric blurring, absorption and thermal emission, but not over large fields of view. Larger ground telescopes, current and planned, play complementary roles in wide-field imaging and multi-object spectroscopy, and in high-resolution imaging to their sharper diffraction-limited resolution—within the contrast and field limitations of adaptive optics. In this section, we outline the capabilities of these current and planned telescopes which form the context for a future large space telescope, which could be built on the Moon or orbited at L2. We consider some details of pixel size and sampling of diffraction-limited images, important aspects in the optical design of the new wide-field telescope.

### (a) Space telescopes

**Figure 1.**
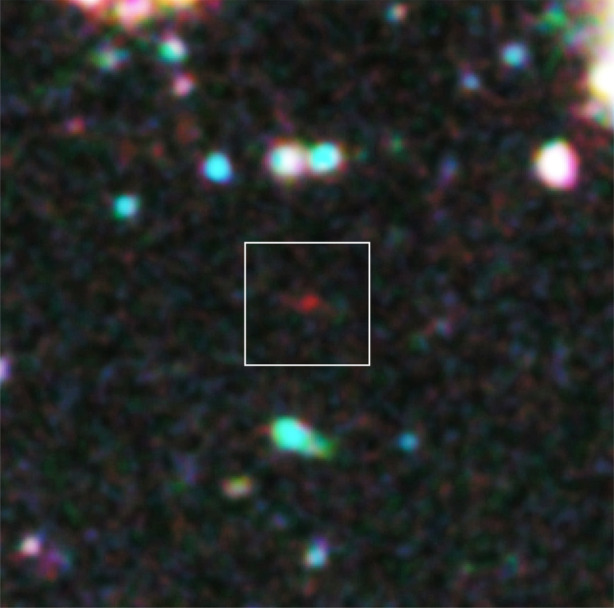
Detail from the HST Extreme Deep Field. The small square is 1.6 arcsec on a side. (Online version in colour.)

The 2.4 m Hubble Space Telescope (HST) has been in operation for 30 years since 1990 and has benefitted enormously from servicing by astronauts. The extreme deep field, for example (figure 1), was obtained by combining images from 0.3 to 1.1 µm wavelengths obtained by the Advanced Camera for Surveys (ACS) installed in 2002 with those from 1.1 to 1.6 µm from the Infrared Wide Field Camera 3 (WFC3/IR) installed in 2009, when the ACS was also repaired [1].

These new instruments took advantage of detector technology advances made since the launch of the telescope, for example, the 1-megapixel HgCdTe imaging sensor used in WFC3/IR. This was used to obtain infrared images over a 2.0 × 2.3 arcmin field of view, a size set by the telescope architecture which allocates different sections of the field of the same size to different instrument slots. Matched to this field at f/11, the HgCdTe detector with 18 µm pixels provided 0.12 arcsec pixel sampling. The shorter wavelength images were recorded over a 2.2 arcmin square field of view with the ACS camera, using a 16-megapixel silicon CCD array at f/26. Here, the 15 µm CCD pixels provide 0.05 arcsec pixel sampling. The high resolution of the Extreme Deep Field, with FWHM close to the pixel sampling size, was obtained by sub-pixel image drizzling or dithering [2].

The Roman Space Telescope (formerly WFIRST) is a second 2.4-m telescope set for launch in 2025 [3]. Its much larger 0.28 square degree field of view is to be imaged by HgCdTe arrays with 0.11 arcsec pixels, providing resolution and sensitivity in the near infrared like that of WFC3/IR. The telescope will operate at 265 K. Wide-field imaging from space will be provided also by Euclid [4], set for launch in 2022, with 1.2-m aperture, and a 0.5 square degree field of view imaged by large CCD and HgCdTe imaging array detectors with, respectively, 0.1′′ and 0.3′′ pixels. This telescope will operate at 130 K.

The 6.5 m James Webb Space Telescope (JWST), currently scheduled for launch in 2021, will be in a distant orbit at L2, as will Roman and Euclid. The telescope is configured for observations into the thermal infrared with a thermal shield for radiative cooling to an operating temperature of 40 K. It is not designed to be serviced, and has a projected lifetime of 10 years, set by the expenditure of fuel to stay on orbit. The JWST instruments, like those of the HST, will each be allocated a small fixed section of the telescope's field of view. Thus, separate infrared cameras image in different wavebands, each sampling a separate 2.2′ × 2.2′ field. Both cameras use a 16-megapixel HgCdTe array with the same 18 µm pixel size as the HST. For the shorter waveband 0.6–2.5 µm, the plate scale is chosen for a pixel resolution of 0.032 arcsec per pixel. JWST provides also for simultaneous spectroscopy over the same waveband of 100 objects, selected by micro-shutters from a 9 square arcmin field of view. The total field of view observed simultaneously by all the JWST instruments is 25 square arcmin.

A concept for a larger successor to HST and JWST now being developed by NASA, for launch in the late 2030s, is the Large Ultraviolet Optical Infrared Surveyor [5]. LUVOIR A, with 15 m aperture, would be placed in a similar orbit in L2 as JWST. As for both HST and JWST, the different instruments would each be apportioned a small fixed 2 × 3 arcmin region of the telescope's field of view. The shorter wave channel would cover the range 200–1000 nm with resolution 0.003 arcsec/pixel, and a near-infrared (NIR) channel will image over the range 1000–2500 nm at 0.008 arcsec/pixel. The information above on pixel sizes for different space telescopes is summarized in table 2 below.

### (b) Ground telescopes

The largest current ground-based telescopes, and the coming generation of 30-m class Extremely Large Telescopes (ELTs) complement space telescopes, with their advantages of greater light-gathering power, and higher diffraction-limited resolution over fields of tens of arcseconds when used with adaptive optics. In addition, moderately sized ground telescopes have opened new scientific vistas by implementing multi-object spectroscopy and imaging over a wide field of view. The 2.5 m Sloan Digital Sky Survey Telescope (SDSS) with a 3° field of view has operated for the past 20 years using separate instruments for imaging and spectroscopy of 1000 objects simultaneously, each using the full field of view [6]. The 8.4-m Rubin imaging telescope (formerly the LSST) will shortly start operation with a 3.2 gigapixel silicon imager array, sampling a 3.5°, 0.64-m diameter field with 0.2 arcsec (10 µm) pixels [7]. Spectroscopic follow-up will use the 3.8-m Mayall telescope now equipped with the DESI multi-object spectrograph, which obtains simultaneously spectra of 5000 objects chosen from a 3.2° field of view [8]. Larger telescopes for multi-object spectroscopy are being designed, for example, 6.5-m and 20 m telescopes with 3° field to accommodate 10 000 positionable 1.2 arcsec fibres [9].

### (c) Lunar telescopes

**Figure 2.**
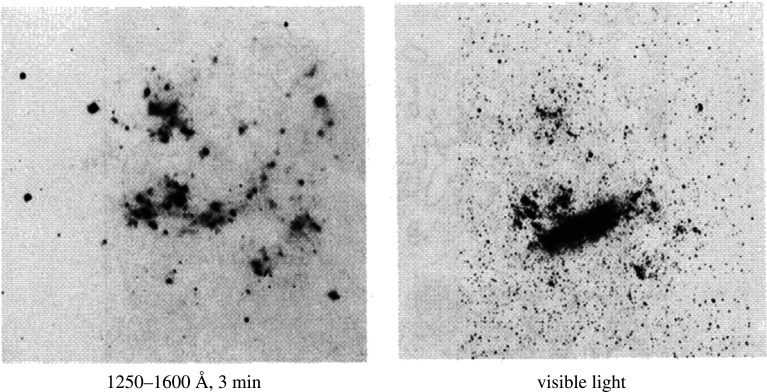
The Large Magellanic cloud imaged with a lunar telescope.

Lunar based telescopes have been designed in the past primarily to exploit the lack of atmosphere mainly for observations in the ultraviolet and for freedom from an atmospheric aberration in coronagraphic observations of exoplanets. Indeed, one telescope made by George Carruthers was operated on the moon by Apollo 16 astronauts in 1972 [10]. A 75 mm vacuum UV Schmidt camera with a 20° field of view, it was used to make a sky survey, including the image of the Large Magellanic cloud shown in figure 2. Several concepts for UVOIR and radio telescopes have been proposed, as considered in detail in a 2007 study [11] and a recent review [12]. These include a spinning liquid mirror of 20–100 m diameter near the lunar pole that takes advantage of Moon's gravity to make a zenith-pointing, cooled, telescope [13]. A spin cast, rigid and steerable telescope mirror as large as 50 m from epoxy loaded with carbon nanotubes and regolith dust has also been considered [14], as has a 1 m class lightweight telescope using a lightweight rigid mirror brought from Earth [15].

### (d) Combining wide field and diffraction-limited imaging in a 20 m space telescope

A uniquely powerful large space telescope might be built if the advantages exploited by JWST and LUVOIR—sharp images and low thermal background—could be augmented with a wide field of view. Imaging and multi-object spectroscopy at the very high diffraction-limited resolution of 20 m aperture would greatly increase the scientific potential for cosmology, especially if the telescope were cryogenically cooled for high infrared sensitivity. Already the wide-field imaging and spectroscopic capabilities of Roman and Euclid will be exploited to better quantify dark energy and dark matter, through baryon acoustic oscillations and weak gravitational lensing for example. But the much larger telescope described here would enable cosmological studies to be taken to a whole new level, allowing direct and detailed observation of the high redshift universe at the time when the first stars and galaxies were forming.

In the following, we explore the optical and technical constraints and show how such a 20 m telescope could be built for diffraction-limited imaging over a 1° field of view, matched to silicon and infrared arrays to cover wavelengths from less than 250 nm to 5 µm. Our hope is that showing such extraordinary technical potential will stimulate thinking by astronomers about the newly enabled science.

## 2. Telescope design

### (a) Design constraints

Previous optical designs for large reflecting space telescopes have not been directed to large fields of view. Our goal is to design a large diffraction-limited field telescope that delivers wide field, to be imaged directly by different very large sensor arrays covering the wavelength range from 250 nm to 5 µm, with no requirement for further optical relays. Similarly, we envisage that multi-object spectroscopy will be via fibres located directly across the full field. This leads to requirements that:

1. The telescope's image scale must be directly matched to image sensors. Pixel size must scale in proportion to wavelength if good sampling of the diffraction-limited image is to be obtained across the broad wavelength range.
2. Full baffling must be incorporated within the main telescope optics.
3. Only reflecting elements to be used, to ensure broad wavelength coverage.

We will suppose that the optical silicon imager sensor to be used in the first generation of instruments covering the 250 nm to 1 µm wavelength range will use BSI-CMOS sensors with approximately 2 μm pixels. Already commercial devices such as the 6-megapixel Sony BFS-U3-63S4C-C are made with 2.4 µm pixels and 2.4 e-read noise. We will suppose also that the 1–5 μm imager will use HgCdTe infrared imaging arrays with 5-μm pixels, as already demonstrated by DARPA [16] imagers. We expect that for future instrument upgrades silicon imagers with 1 µm pixels, a size already used in cell phones, will become available with very low readout noise for the blue and UV spectrum. Similarly, we envisage that infrared arrays with 2.5 µm pixels may become available on the timescale of decades before second generation instruments are built.

The focal length for the telescope is chosen such that an initial instrument suite using imagers with optical and infrared pixel sizes of 2 and 5 µm will sample the very sharp 20 m diffraction-limited PSF with pixels the same size as the airy core, as in the HST ACS and WFC3/IR cameras. The same dithering methods will be used to recover close to the diffraction-limited resolution 1.22 λ/*D* down to a minimum wavelength λ_min_ at which the pixel size = λ_min_/*D*, i.e. 500 nm for the silicon imager and 1.25 µm for the infrared. These are essentially the same minimum wavelengths as for the HST; thus, the images will be like the Extreme Deep Field broadband image of figure 1, *except with 8 times higher resolution*. Future upgrades halving the pixels sizes would then also halve these minimum wavelengths to 250 and 625 nm.

On this basis the required focal length is then 80 m, i.e. a focal ratio for the 20 m diameter telescope is f/4 and the plate scale is 360 µm/arcsec. Fortuitously, f/4 is close to the focal ratio of f/3.7 used to couple into fibre optics. Table 1 summarizes the pixel sizes in micrometres and arcseconds implemented in the HST future space telescopes and targeted for the wide-field imagers in the 20 m telescope at launch (1) and for later instrument updates (2).

**Table 1.** Pixel sizes and minimum wavelength for different space telescopes.

		pixel size				wavelength for 1 pixel = λ/*D*	
telescope	diameter (m)	Si	HgCdTe	Si	HgCdTe	Si	HgCdTe
HST	2.4	15 µm	18 µm	50 mas	120 mas	580 nm	1400 nm
JWST	6.5	—	18 µm	—	32 mas	—	1000 nm
LUVOIR	15	6.5 µm	10 µm	3.7 mas	7.4 mas	270 nm	540 nm
EUCLID	1.2	12 µm	38 µm	100 mas	320 mas	580 nm	1750 nm
Roman	2.36	—	10 µm	—	110 mas	—	1260 nm
20 m (1)	20	2.0 µm	5.0 µm	5.2 mas	13 mas	500 nm	1260 nm
20 m (2)	20	1.0 µm	2.5 µm	2.6 mas	6.5 mas	250 nm	630 nm

An additional important design consideration comes from coronagraphic imaging of exoplanets, namely central obscuration. This should be minimized in order to minimize starlight diffracted outside the central diffraction peak of the star. For the Airy pattern of a fully filled aperture, the rings amount to 14% of the total. We target keeping the central obscuration below 10% by area, in order to no more than double the starlight halo in diffraction rings to be suppressed by the coronagraph. As further practical constraint, we have set an upper size limit of around 6 m diameter for the secondary and subsequent optical elements, to limit central obscuration.

### (b) Design approach and method

Three-mirror and four-mirror anastigmats have been adopted or previously investigated to obtain sizable fields of view that are diffraction-limited at optical wavelengths. A three-mirror system was chosen for the 39 m diameter ELT. It realizes a 10-arcmin field of view at f/18, with wavefront errors less than 15 nm RMS [17]. A three-mirror anastigmat has been investigated also for the 15-m aperture LUVOIR A, in an off-axis design optimized for small central obscuration, 10% in radius [18]. It achieves an average wavefront error of 6 nm RMS over an 8 × 10 arcmin field of view.

With the objective of obtaining substantially larger, diffraction-limited field of view, we have explored axisymmetric designs using four powered mirrors. This configuration is very favourable in that no fold mirrors or added correcting optics are required in order to obtain a very wide field with diffraction-limited imaging. An additional advantage is that the only optical losses are those that take place at the four powered mirrors, and at the bandpass filters used in wide-field imaging. The primary and secondary mirrors (M1 and M2) take the form of a two-mirror telescope forming an intermediate focus located in the centre hole of a quaternary mirror (M4). The final image is formed at an accessible position through the centre hole in the tertiary, M3 [19]. This configuration provides greater potential to achieve our design goals. A design of this type targeting diffraction-limited fields of 10 arcmin for multi-conjugate adaptive optics was developed for a 40-m ELT concept by Brusa *et al.* [20] and Goncharov [21] with diffraction-limited images in visible light over an 8 arcmin field of view at a focal ratio of f/12. The main factor limiting the usable field of view with this type of design is internal vignetting, which occurs on the rims of the central holes of M3 and M4. The design yielded obscuration for an axial beam of 0.31-m in radius, resulting in 9% light loss.

In exploring the limits of four-mirror designs of this type to obtain wide-field imaging, we can take advantage of faster focal ratios at both the intermediate and final foci to reduce the physical diameter of the field, and thus to limit such central obscuration. M3 and M4 are also made as large as possible, to the limit set by their central obscuration within the primary telescope aperture. Designs to balance the above constraints while minimizing wavefront aberrations have been explored using Zemax OpticStudio.

### (c) Preferred design with 1° field of view

**Figure 3.**
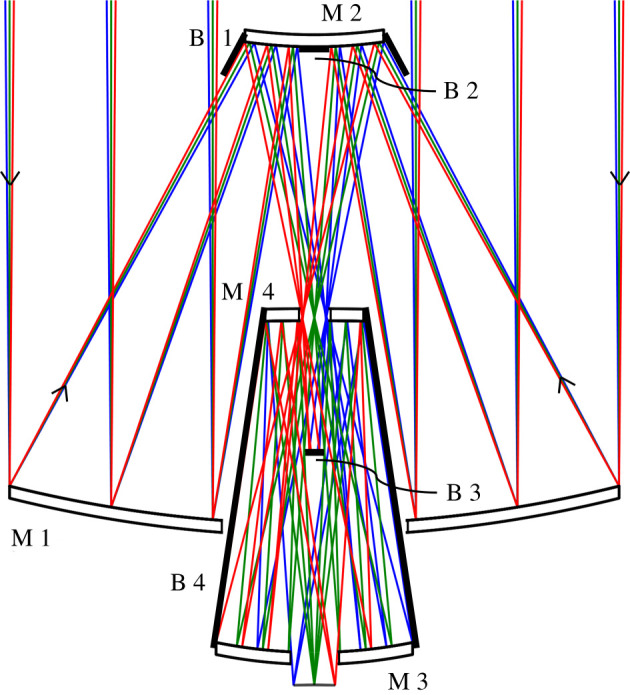
Design for a 4-mirror 20 m telescope with an unvignetted and fully baffled 1° field of view. (Online version in colour.)

Here, we show our most promising design for a 20 m telescope, obtained using four axisymmetric mirrors and yielding a 1° diameter field of view at f/3.9. The ray diagram is shown in figure 3, and the optical prescription is given in table 2. The 20 m f/1.0 primary M1 is configured with a 4.6-m convex secondary M2 to obtain an intermediate focus at f/3.1, where a field of 1° passes through a hole 0.92-m in diameter in a 3.2-m diameter quaternary M4. A 6.5-m diameter tertiary mirror M3 set 5 m behind the primary forms a 3.0-m exit pupil on M4. Light reflected by M4 passes through the primary and a 1.5-m diameter hole in M3 to a 1.36-m diameter, 1° focus at f/3.9. In this way, the full field is unvignetted, and the on-axis central obscuration is held to 9% of the full area of the primary mirror.

**Table 2.** Optical prescription. Dimension in metres.

element	radius	thickness	outer diameter	inner diameter	conic	P-V aspheric departure (mm)
primary	−40.000	−15.819	20.000	6.000	−0.848	4.35
secondary	−15.497	13.383	4.547	—	−4.883	1.08
baffle	flat	6.700	0.600	—	0.000	—
tertiary	−15.839	−11.000	6.472	1.520	−0.575	0.49
quaternary	80.280	12.080	3.236	0.920	0.000	0.14
image	13.832		1.365	—	0.000	0.02
aspheric terms	4th order	6th order	8th order	10th order	12th order	14th order
primary	—	3.416 × 10^−10^	2.373 × 10^−12^	−3.699 × 10^−14^	2.002 × 10^−16^	−4.230 × 10^−19^
secondary	—	8.995 × 10^−7^	−4.121 × 10^−8^	−4.528 × 10^−10^	−4.122 × 10^−10^	5.529 × 10^−11^
tertiary	—	−1.283 × 10^−7^	2.555 × 10^−8^	−3.145 × 10^−9^	2.138 × 10^−10^	−6.025 × 10^−12^
quaternary	−1.684 × 10^−4^	2.260 × 10^−5^	6.644 × 10^−6^	−4.605 × 10^−6^	1.086 × 10^−6^	−9.906 × 10^−8^
image	−2.848 × 10^−4^	—	—	—	—	—

**Figure 4.**
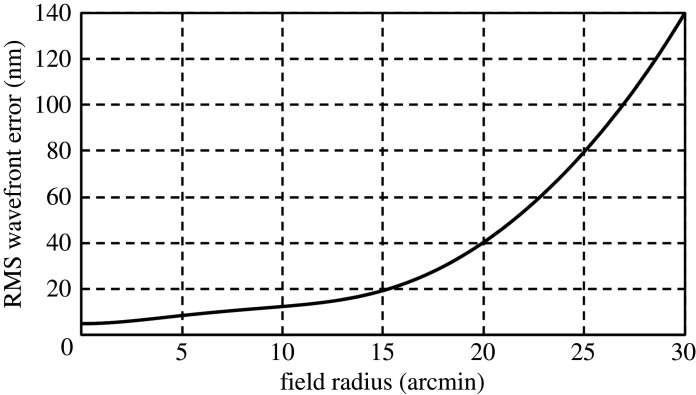
Wavefront aberration as a function of field angle.

**Figure 5.**
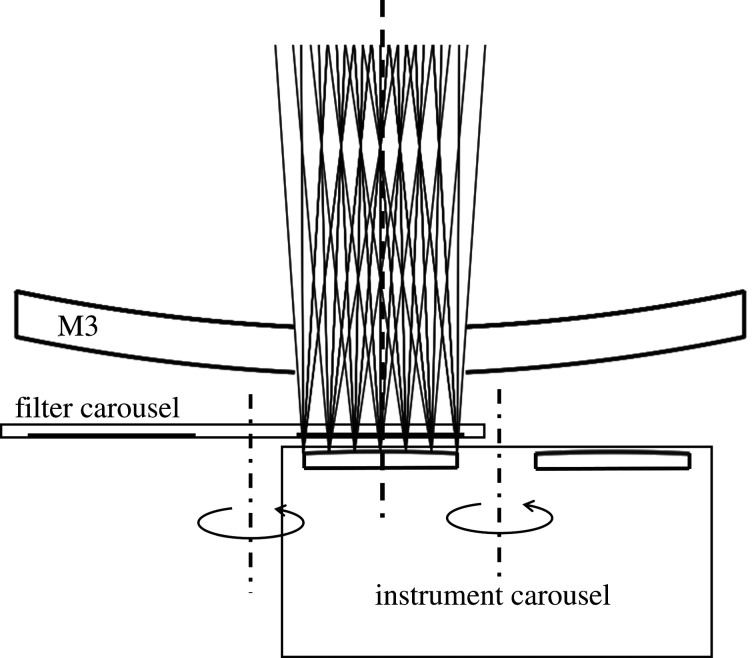
Carousel mounted below the 6.5 m tertiary mirror carries five instruments, each with access to the full 1° field of view. Bandpass filters are mounted on a separate carousel.

Wavefront aberration, as shown in figure 4, is 5 nm RMS at the field centre, increasing to 140 nm at 30 arcmin field radius. The entire field of view is diffraction limited (Strehl ratio greater than 80%) for wavelengths of 2 µm and longer. At 1 µm wavelength, the diffraction-limited field of view extends out to 50 arcmin diameter (wavefront error less than or equal to 70 µm RMS), and at 500 nm wavelength over a 40-arcmin diameter. The image formed in the ultraviolet at 280 nm wavelength is diffraction-limited over a half-degree field of view, where the RMS wavefront error is better than 20 nm RMS. Figure 5 shows different instruments including optical and infrared imaging arrays mounted on a carousel at the focus, each having access to the full 1° field.

The field of view is fully baffled at all field angles. Stray ray paths from the sky directly or by mirror reflections to the image surface are completely blocked by the four baffles shown in figure 3. Two conical baffles B1 and B4 are located around the secondary mirror and between M3 and M4. An axial circular baffle B3 0.6-m in diameter is located between the primary and secondary mirrors to block a direct reflection path from the secondary to the image plane. Additionally, a circular baffle B2 is located at the centre of the secondary mirror preventing multiple mirror reflection paths to the imaging plane. The conical baffle B4 extending from M3 to M4 will be made with blackened annular rings to prevent light from the sky entering directly through the M4 aperture and scattering from the baffle onto the image surface. The baffling performance was verified in a non-sequential reverse ray trace where rays were traced leaving at all angles from the image plane out of the baffled telescope. The radiant intensity on the sky was bounded within ±0.5°, showing that only light entering over the 1° field of view will reach the image plane.

#### (i) Alignment tolerance and active wavefront correction

In order to realize in practice, the full potential of this design, active wavefront sensing and control and metrology subsystems will be used to compensate for errors in segment alignment (piston and tip/tilt), secondary mirror alignment, and global low-order wavefront errors. A detailed tolerance analysis is beyond the scope of this paper, but a concept for such systems has been made for the similarly sized 15-m LUVOIR A. For on-axis coronagraphic imaging, it uses small auxiliary active mirrors in the coronagraph instrument optics [22]. Our design presents additional challenges in order to maintain diffraction-limited imaging over a much wider wide field of view. Furthermore, if sited on the Moon with 1/6 Earth's gravity, discontinuities at the segment edges can be expected to vary as a function of elevation angle and varying thermal gradients.

Our four-mirror design is well adapted to active correction over a wide field of view, because of our location of the relatively small M4 at a position conjugated to the primary mirror. Actively controlled deformation of this mirror at the exit pupil can thus be used to correct wavefront errors arising at the primary M1, over the full field of view. The active M4 mirror may be segmented in the same patterns as the primary, so that segment edge errors may be accurately corrected, as well as low and medium order figure errors.

The active M4 mirror may also be used to correct other misalignment errors. Suppose, for example, that the secondary mirror M2 is displaced laterally by 100 µm, as might happen by gravitational deflection with a change in elevation. A large comatic wavefront error of approximately 280 nm RMS will result. By making a correction at M4, and adjusting the mirror spacing, this error can be reduced to an increase in the RMS error of no more than 13 nm at any radius over the ideal error shown in figure 4. For coronagraphic imaging, there would be an 8 nm increase in the on-axis wavefront error, which would be corrected by the coronagraph's active system. We conclude that by using the kinds of sensing and correction envisioned for LUVOIR, plus the large scale (3 m) active M4 mirror, it is realistic to achieve the full potential for wide field and coronagraphic imaging.

#### (ii) Image PSF

**Figure 6.**
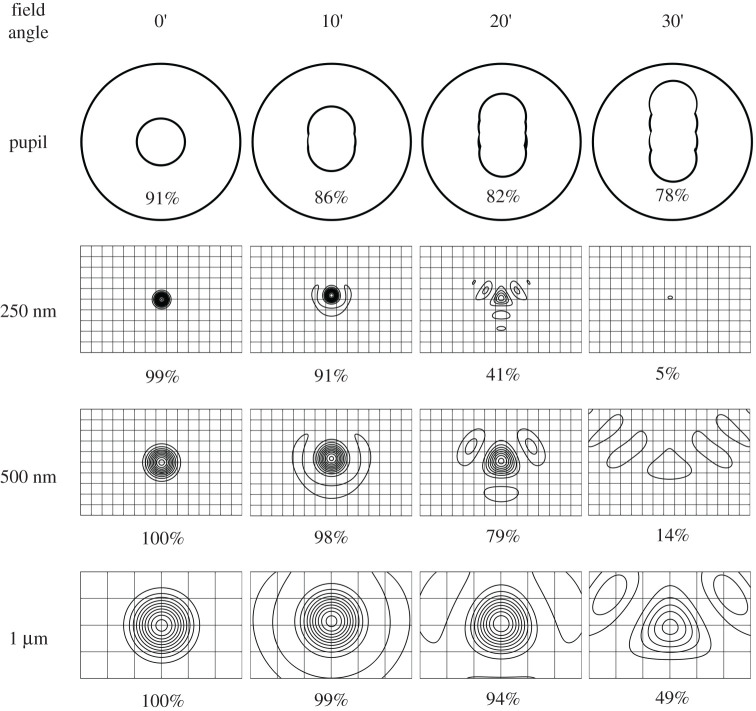
Pupil obscuration as a function of field angle (*a*) and point spread functions (psf) as a function of field angle and wavelength. The rectangular frames cover 10 × 15 µm, with a 1.0 µm grid shown for 250 nm (*b*) and 500 nm wavelengths (*c*), and a 2.5 µm grid for µm wavelength (*d*). The percentages in row (*a*) are vignetting and those in rows (*b*–*d*) are Strehl ratios.

**Figure 7.**
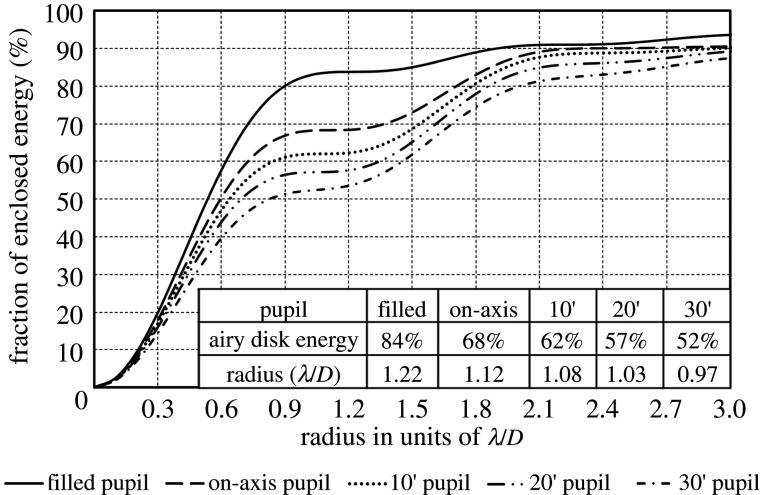
Dependence of encircled energy on central obscuration for the unapodized pupils shown in figure 6 at different field angles, assuming negligible or corrected wavefront aberration. Also shown is the encircled energy for a filled pupil (Airy pattern).

A more complete picture of the image and how it would be sampled by imaging arrays with different pixel sizes is provided in figure 6, which shows as a function of field angle the central obscuration (figure 6*a*). The diffraction point spread functions (psfs) calculated at wavelengths of 250, 500 and 1000 nm in figure 6*b–d* show the combined effects of wavefront error and central obscuration. The contours are at 10% intensity intervals, from 5% to 95%, with the intensity normalized to the peak intensity for the same pupil obscuration but un-aberrated wavefront. The absolute peak intensities are also provided in figure 7. The psfs at 250 and 500 nm wavelength are shown against a 1.0 μm grid, and for 1 µm wavelength against a 2.5 µm grid, representing the resolution projected for high resolution optical and infrared imagers.

Central obscuration moves some light from the central diffraction peak into the diffraction rings, even with no wavefront aberration. This effect is quantified for the obscuration in the 20 m telescope at different field angles in figure 7, which shows encircled energy as a function of image radius for un-aberrated or actively corrected wavefronts. Also shown is the encircled energy for a completely unobscured pupil, the classic Airy pattern, where the first shoulder is at radius 1.22 λ/*D* with 16% of the energy outside the peak and in diffraction rings. For our telescope on axis, the effect of 9% central obscuration is to shrink the first dark ring to radius 1.12 λ/*D*, and to double the energy in the diffraction rings outside this ring to 32%.

For exoplanet detection, strong suppression of the stellar diffraction halo is required. For this reason, the current design concept for the 15 m LUVOIR A, [5], adopted a primary mirror with a central hole blocking only 1% of the primary aperture, halving the diffraction halo compared to the design we propose, but also eliminating the potential for wide-field imaging. Fortunately, coronagraphs using phase-induced amplitude apodization can strongly suppress stellar diffraction from central obscuration as well as from the sharp outer mirror boundary and segmentation, while achieving an inner working angle close to the λ/*D* limit [23]. Thus, the present design can be powerful for on-axis direct imaging of exoplanets as well as for wide-field imaging.

## 3. Manufacture, structure and operation on the Moon

If a 20 m telescope of the type described above were to be located on the Moon, near a human outpost, then, like the Hubble telescope, it could benefit from astronaut repairs and upgrades over decades of operation, including replacement of silicon detectors which are degraded by cosmic rays in space. New technology and instrumentation would be directed to new scientific priorities.

What kind of telescope architecture best suited? We envisage a telescope built from large subassemblies, sized to fit into a launch vehicle for assembly on the Moon, where orientation would be by geared motors in an alt-azimuth mount, as for large telescopes on Earth. This is quite different from the architecture of the 6.5-m JWST, and proposed for the 15 m LUVOIR A. These designs are engineered for completely remote deployment and operation at L2, for maximum weight reduction, and for primary mirrors made from many separate small segments, 18 for JWST, 120 for LUVOIR A.

In this section, we touch on some of the features of a telescope whose architecture is optimized for construction and operation taking advantage of the infrastructure, robotic and human, already associated with a lunar habitat. Also, in anticipation of future larger payload masses, we consider designs using robust mirror subassemblies matched to the capacity of projected future vehicles for lunar development. These would be transported in a stack, and then lifted off one by one for assembly on site.

### (a) Mirror subassemblies

**Figure 8.**
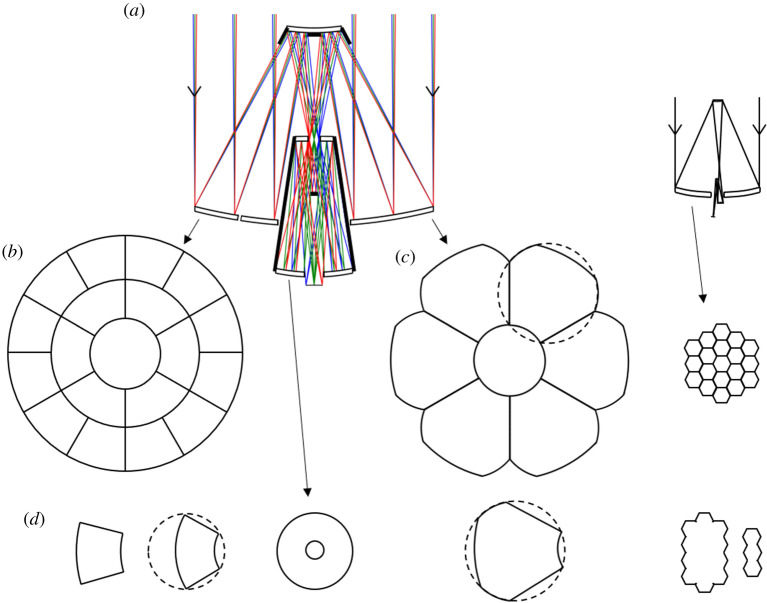
(*a*) Optical layouts drawn to scale for this telescope and JWST (right). Options for using 18 or 6 primary mirror segments are shown in (*b*) and (*c*). (*d*) shows the 6.5 m tertiary and 20 m segment subassembly options in plan view, as they would be stacked into payloads of 6.5-m or 8.4 m diameter, and the JWST mirror subassemblies.

We envisage the 20 m primary mirror taking the form of either 6 or 18 subassemblies, as shown in figure 8. Figure 8*b* shows it made with 18 trapezoidal assemblies. The six inner ring segments each fit into a 6.5-m circle, as shown in figure 8*d*, and all the segments along with the 6.5-m tertiary would be stacked for launch in a 6.5-m diameter payload. Figure 8*c* shows an alternative with just six identical petal-shaped subassemblies, each fitting in an 8.4-m diameter circle. These would be stacked to fit within the 9-m diameter limit of the SpaceX Super-Heavy launch vehicle. (The slightly incomplete outer boundary in this segmentation increases the on-axis diffraction rings flux by 7%). For comparison, figure 8 shows to scale on the right the JWST, whose 6.5-m primary mirror is made in three subassemblies, hinged and folded about a vertical axis for launch.

The 20 m segments could be made as monolithic glass mirrors, like the segments of the Giant Magellan Telescope primary, being made at the University of Arizona Richard Caris Mirror Laboratory. One of the six 8.4-m diameter, off-axis segments of the f/0.7, 25-m primary mirror is already finished to 14 nm RMS surface error, the specification for this ground-based telescope [24]. For the 20 m primary segments, the higher tolerance less than 10 nm surface error targeted is within the capability of the currently proven methods of metrology and figuring.

While the GMT honeycombed glass mirrors weigh 290 kg m^−2^, too heavy for launch, recent studies show that lighter weight glass mirrors suitable for space can be made, with specific masses well under the 180 kg m^−2^ of the HST fused silica honeycomb mirror. For example, the University of Arizona has analysed the potential for a 4-m honeycombed borosilicate glass mirror with specific density 95 kg m^−2^ [25]. At this density, the six primary mirror segments would weigh a reasonable total of 30 tons. To ensure launch survival and accurate optical figure in use, the segments would be mounted as subassemblies in carbon fibre mirror cells.

### (b) Thermal shield at the Moon's south pole

The telescope will be on an alt-azimuth mount, rotating in azimuth once a month about the polar axis, which is nearly vertical. There is then no field rotation. Cooling the telescope to 100 K or less at the lunar pole for infrared work requires it to be shielded from sunlight, which comes only from close to the horizon. While sitting in a crater has previously been suggested [13], we propose siting on level ground with shielding by a deployable lightweight cylindrical surround. This has the advantage that it may be lowered to warm the telescope during construction, servicing or upgrades. A screen around the telescope may also be advantageous to reduce dust contamination.

**Figure 9.**
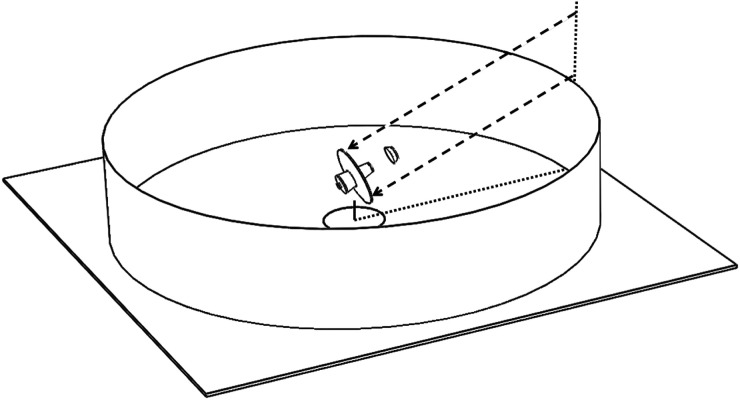
20 m telescope at the lunar pole with a cylindrical sunshade. Rays drawn for 20° elevation.

Figure 9 illustrates the telescope surrounded by a cylindrical thermal shield. This is made large enough that observations are unobscured down to 20° elevation, allowing access to 1/3 of the full sky, and high enough to shade the high secondary mirror and baffle from sunlight when the telescope is zenith pointed. The sun never rises more than 1.5° above the horizon, because the moon's spin axis is nearly perpendicular to the ecliptic plane. The Earth rises to 5° above the horizon. Setting the telescope's elevation axis to be 15 m above flat ground, the shield must be 80 m in radius and 33 m high, with an area of 16 600 m^2^.

We envisage the shield made from cylindrical curtain arc sections hanging from two concentric circular rails. To admit sunlight when the telescope is to be serviced, the sections can be rotated past each other to open a gap. If made like the JWST sunshade from five layers of aluminized kapton film, the curtain material would weigh a total of 3.5 tons. During local ‘summer’, when the sun is at its highest elevation, the upper 6 m of the curtain across from the sun will be illuminated on the inside. Here, the ideal surface finish would be vapour deposited silver, which has an absorptance of 4%. The curtain surface would be imprinted like a Fresnel lens to reflect the sunlight up by a few degrees, away from the telescope and out of the cylinder. If required, the lunar surface within the cylindrical wall could be treated to eliminate the release of dust.

## 4. Discussion and conclusion

We have shown that a 20 m telescope in space, free from atmospheric blurring, could be made using four axisymmetric mirrors to have diffraction-limited images over a uniquely large 1° field of view, at a relatively fast focal ratio of f/3.9. The big field of view is fully baffled and is readily accessible behind the quaternary mirror. Central obscuration is limited to less than 10% in area, as is desired for coronagraphic exoplanet imaging. The focal ratio is slow enough to allow for direct imaging by full-field arrays of optical or infrared imagers with small pixels, with no requirement for fold mirrors or additional relay optics. BSI-CMOS and HgCdTe arrays with pixel sizes of 2 and 5 µm, respectively, have been demonstrated, corresponding at the f/3.9 focal ratio to λ/*D* sampling at wavelengths λ of 500 nm and 1.26 µm. For λ/*D* sampling in the ultraviolet, 1 µm pixel detectors are already common, and are likely to be available at the size and quality needed in time for this telescope. The f/3.9 focal ratio is also well matched to feed fibres for multi-fibre spectroscopy.

The design could be implemented in a lunar telescope, the subject of these proceedings, or in a telescope in free space, with 20 m aperture, or scaled down to 15 m. It thus offers for LUVOIR an alternative design from the three-mirror anastigmat now being studied, which delivers fields of view of only a few arcmin to fixed instruments via folding and relay optics. Our design, with a full 1° field of view accessed directly by each of several different carousel-mounted instruments allows for a broader range of scientific investigation, for cosmology.

If the telescope were to be built at a lunar pole, accessible from an already established long-term human outpost, its construction and operation would be greatly facilitated by taking advantage of the infrastructure already in place. As the HST has shown, a space telescope used over several decades, with occasional upgrades as the science and technology advance, is very attractive. Implementation of the design on the Moon could be more like that of the coming generations of 25–40 m ground-based telescopes than the automatically unfolded JWST. The telescope could be delivered to the Moon in the form of a stack of large optical and mechanical subassemblies. Unloading and assembly could use habitat equipment already in place, for example, that used to construct a habitat/greenhouse from locally sourced materials, as envisaged by Woolf and Angel in these proceedings [26]. The primary mirror could be constructed from 6 or 18 large segment assemblies, made by optical manufacturing methods already largely proven at the scale and accuracy required.

From the above considerations, it seems well worthwhile to explore the added scientific potential provided by wide-field imaging and spectroscopy of this design for a future very large space telescope like LUVOIR. We have touched here on some of the key engineering issues for lunar siting, but many remain to be addressed. A more detailed design analysis and cost projection for building the telescope, either in free space or adjacent to a lunar polar habitat would be valuable, as would a quantitative study on maximizing coronagraphic performance consistent with the wide-field imaging capability.

## Data accessibility

This article does not contain any additional data.
